# When cell teamwork turns toxic

**DOI:** 10.7554/eLife.106689

**Published:** 2025-04-10

**Authors:** Wadih EI Khoury, Stephen Y Chan

**Affiliations:** 1 Center for Pulmonary Vascular Biology and Medicine, Pittsburgh Heart, Lung, and Blood Vascular Medicine Institute, Division of Cardiology, Department of Medicine, University of Pittsburgh, School of Medicine and University of Pittsburgh Medical Center Pittsburgh United States

**Keywords:** pulmonary artery, adventitial fibroblasts, vascular smooth muscle cells, pulmonary hypertension, Human

## Abstract

In pulmonary hypertension, a combination of metabolic and mechanical dysfunction leads to irreversible vascular damage.

**Related research article** Crnkovic S, Puthenparampil HT, Mulch S, Biasin V, Radic N, Wilhelm J, Bartkuhn M, Rad EB, Wawrzen A, Matzer I, Mitra A, Leib R, Nagy BM, Sahu-Osen A, Valzano F, Bordag N, Evermann M, Hoetzenecker K, Olschewski A, Ljubojevic-Holzer S, Wygrecka M, Stenmark K, Marsh LM, de Jesus Perez V, Kwapiszewska G. 2025. Adventitial fibroblasts direct smooth muscle cell-state transition in pulmonary vascular disease. *eLife* **13**:RP98558. doi: 10.7554/eLife.98558.

Cells rely on cues from their environment to develop and work properly. Yet this interdependence can turn perilous when one cell goes rogue, triggering an unchecked cascade of dysfunction. For decades, understanding these toxic cellular relationships has been hindered by a fundamental challenge: the inability to simultaneously study molecular details and tissue-wide interactions. Earlier studies often prioritized one scale at the expense of the other and, because of this, missed critical connections.

A striking biological example is the cellular interplay in pulmonary hypertension. This life-threatening disease, defined by an increased pressure in the blood vessels of the lungs, often ultimately leads to heart failure and death (Humbert et al., 2019). At its roots, pulmonary hypertension arises from the toxic interaction between multiple cell types compromising each other’s normal function (Frid et al., 2020). Under healthy conditions, smooth muscle cells in the arteries of the lungs (known as PASMCs) contract and dilate, regulating flow and pressure in the lung vasculature. Their counterparts, the fibroblast cells that form the vessel’s outer layer (or PAAFs), protect PASMCs and mediate communication between the two cell types (Stenmark et al., 2018).

In pulmonary hypertension, however, this partnership turns lethal. PAAFs start remodeling their external environment and secrete signals that cause PASMCs to lose their ability to contract, depriving them of their main function (Park et al., 2022). Instead, the PASMCs start stiffening, causing a cascade of irreversible vascular changes that represent a cornerstone in the development of pulmonary hypertension (Crnkovic et al., 2022).

With PAAFs and PASMCs at the forefront of this disease, many questions remain unanswered. What triggers PAAF activation? How do PASMCs lose their contractility? Is there an underlying shared mechanism linking PAAF and PASMC dysfunction? Crnkovic et al., 2022 Answering these questions could lead to new and more effective therapies for a number of deadly diseases.

Now, in eLife, Grazyna Kwapiszewska, Vinicio de Jesus Perez and colleagues at various research institutes in Austria, Germany and the United States – including Slaven Crnkovic and Helene Thekkekara Puthenparampi as joint first authors – report how PAAFs force PASMCs into a diseased state (Crnkovic et al., 2025).

Crnkovic et al. isolated PAAFs and PASMCs from both healthy donors and pulmonary hypertension patients, comparing the gene activity and protein levels between these two groups. They found that diseased PAAFs produced more collagen, which stiffens tissues, while simultaneously underproducing laminin, a protein that provides structure for collagen deposition (Figure 1). This collagen-laminin imbalance created a rigid environment, similar to a building with too much concrete and not enough scaffolding. The mechanical stress from the stiffened matrix forced PASMCs to abandon their contractile function, a hallmark of their healthy state (Crnkovic et al., 2022).

**Figure 1.**
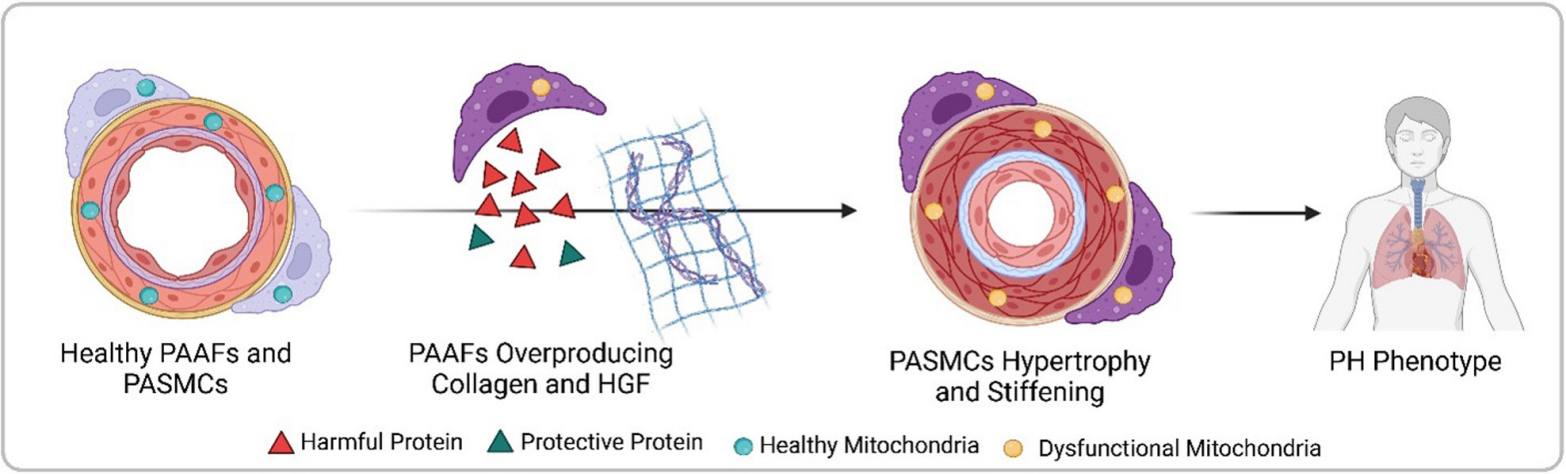
Progression from healthy pulmonary artery adventitial fibroblasts (PAAFs) and pulmonary artery smooth muscle cells (PASMCs) to pulmonary hypertension (PH) phenotype. In healthy arteries (left), PASMCs are dynamic cells that regulate flow and pressure in the lung vasculature by contracting and dilating, while the fibroblasts on the vessel’s outer layer (PAAFs) protect PASMCs (green triangles). In pulmonary hypertension, PAAFs overproduce collagen and harmful proteins (red triangles) that cause PASMCs to become rigid. Imbalanced protein dynamics and mitochondrial dysfunction in both cell types further contribute to the diseased state (right). Created with BioRender.com.

In a parallel mechanism, diseased PAAFs exacerbated dysfunction by overproducing harmful signals while downregulating protective proteins, thus reprogramming PASMCs into a dysregulated state. As dynamic cells require constant energy, PASMCs rely heavily on mitochondria, especially in times of added cellular stress (Qin et al., 2023). Given the central role of mitochondria in generating cellular energy, regulating harmful molecules, and maintaining an adequate intracellular balance – all of which are affected in pulmonary hypertension – Crnkovic et al. investigated the mitochondria’s role in reprogramming PAAFs and PASMCs (Chan et al., 2009).

Next, mitochondrial function was evaluated by measuring membrane potential, oxygen consumption and levels of harmful reactive species, which linked the observed mitochondrial compromise to subsequent DNA damage. This analysis revealed that mitochondrial dysregulation occurred when both cell types transitioned to a diseased state. Mitochondria are crucial in protecting the cell’s DNA by neutralizing harmful molecules, especially in diseased states. However, because the mitochondria were damaged, the DNA suffered additional stress, worsening cellular injury.

In pulmonary hypertension, microenvironment stiffness and mitochondrial dysfunction are two sides of the same coin. The rigid environment forces PASMCs into a state of mechanical stress, overloading their mitochondria. In turn, dysfunctional mitochondria, while unable to protect the DNA, also leak harmful molecules, worsening cellular remodeling. This vicious cycle, where mechanics and metabolism unite, traps the cells in a diseased state leading to irreversible vascular damage. By uncovering these pathways, Crnkovic et al. revealed that targeting PAAF signaling has the potential to reverse PASMC dysfunction in pulmonary hypertension. Halting toxic crosstalk and mitochondrial damage could thus reverse vascular stiffening, a potential therapeutic breakthrough for this disease.

While this study advances our understanding of pulmonary hypertension, certain limitations must be acknowledged. First, by focusing only on samples from patients with an established disease, early markers of disease triggers remain unexplored (Stacher et al., 2012). Second, the study only focused on PASMCs and PAAFs, potentially excluding other significant but understudied cell types in the progression of the condition (El Kasmi et al., 2014). Third, the origin of the reported mitochondrial dysfunction, whether a cause or a consequence of pulmonary hypertension, remains unclear.

By evaluating the interplay between different cell types while pinpointing precise genetic targets, Crnkovic et al. highlight the transformative potential and inherent challenges of studying genes and protein profiles in pulmonary hypertension. Leveraging this approach in future work, researchers may be able to shed light on pulmonary hypertension’s full story and provide a therapeutic road map to restore healthy cell states for a disease with no current cure.
